# Role of exosomes in transforming growth factor-β-mediated cancer cell plasticity and drug resistance

**DOI:** 10.37349/etat.2025.1002322

**Published:** 2025-06-05

**Authors:** Tatiana Ruksha, Nadezhda Palkina

**Affiliations:** IRCCS Istituto Romagnolo per lo Studio dei Tumori (IRST) “Dino Amadori”, Italy; Pathophysiology Department, Krasnoyarsk State Medical University, 660022 Krasnoyarsk, Russian Federation

**Keywords:** Extracellular vesicles, dormancy, quiescence, microenvironment, transforming growth factor-β

## Abstract

Transforming growth factor-β (TGF-β) is a multifunctional molecule with a dual role in carcinogenesis. Recent studies have demonstrated its various effects on cancer-related processes. However, the identification of TGF-β and TGF-β signaling pathway regulators in extracellular vesicles (EVs) appears promising for targeting them to control cancer progression associated with drug resistance. Exosomal TGF-β has been shown to be implicated in cancer cell phenotypic plasticity, a dynamic feature of cancer cells, and an evasive process hampering treatment efficacy. Additionally, EVs can influence the metastatic cascade through mechanisms, including their effects on the immune system and their binding to extracellular matrix (ECM) proteins. These processes collaborate to provide a supportive microenvironment for the development and growth of metastatic tumors. A deeper understanding of the mechanisms by which EVs facilitate TGF-β-mediated intercellular communication may have practical implications for better controlling oncological disorders and providing new methods for cancer diagnostics and treatment, including approaches targeting EVs.

## Introduction

Cell communication driven by extracellular vesicles (EVs) is considered a powerful strategy in tumor progression and dissemination [1]. EVs, including exosomes, are small membrane-bound structures that transport regulatory molecules. Cells produce two distinct types of EVs: microvesicles (MVs) and exosomes. MVs are characterized by a diameter of 0.2–2.0 μm and are generated from membrane-enclosed packages from plasma membranes. Exosomes are smaller than MVs, measuring 0.03–0.1 μm in diameter, and are formed as a result of the endosomal sorting complex required for transport- and Rab-dependent rerouting of multivesicular bodies containing endosomes from the lysosome, where they would be degraded, to the cell surface [2]. The multivesicular bodies then fuse with the plasma membrane and release their contents into the extracellular environment, at which point they are referred to as exosomes [3]. EVs play a crucial role in cell-to-cell communication, impacting basic physiological and pathological processes [4]. Recent research has highlighted the role of EVs in tumor growth and metastasis development. It is well established that tumors can precondition distant organs to create a supportive environment for future metastasis [5]. This priming enables disseminated cancer cells to reside and give rise to secondary cancer cell sites. Only a small fraction, no more than 0.01% of cells, successfully adapt to the altered environment, followed by establishing a distant site of tumor growth [6]. Moreover, once disseminated cancer cells reach favorable conditions in distant organs, they can remain quiescent for extended periods. Quiescent cells are not actively proliferating and are referred to as slow-cycling cells reversibly residing in the G0 phase of the cell cycle. Thus, they escape the effects of standard anti-tumor treatments, which mostly target actively dividing cells. Under less evident stimuli, quiescent cancer cells re-enter the cell cycle to give rise to metastasis. Tumor dormancy is closely related to drug resistance and significantly impacts cancer therapy efficacy [7].

It remains challenging to unveil the precise mechanisms by which quiescent disseminated cancer cells re-enter the cell cycle and start to divide again. This shift from a dormant to a proliferative state is believed to be regulated, at least in part, by signals provided through EVs [8]. Exosomes are released by both tumor and normal cells and can transfer molecular cargo consisting of proteins, lipids, and nucleic acids from host to recipient cells [9, 10]. Some of these molecules, acting as signals, can trigger residing cancer cells to exit quiescence and start to proliferate, ultimately contributing to metastasis and treatment resistance [11]. The relevance of studying distant cell communication via exosomes and other EVs is underscored by its potential to understand tumor progression and implement novel treatment strategies for cancer [12]. Transforming growth factor-β (TGF-β) is considered a multifunctional cytokine with various cancer-related functions. Both TGF-β and its regulators, transferred via exosomes, are implicated in cancer cell plasticity, which is a basis of drug resistance.

The aim of this review is to describe the current status of Evs’ role in TGF-β-mediated cancer cell phenotypic shifting, and the dependence on cell-to-cell communication, as well as cell interaction with the microenvironment through EVs, to better understand the mechanisms that enable tumors to escape treatment and promote growth and progression in distant organs. This data may serve to develop novel approaches for overcoming cancer cell drug resistance, tumor dissemination, and support new strategies for cancer treatment. By focusing on EV-mediated communication, evident insights that pave the way for more effective cancer therapies can be discovered.

## Exosomes and oncosomes

Exosomes were first characterized in 1983 by Pan and Johnstone [13] as small-sized EVs released by a broad spectrum of normal cells, including cells of epithelial origin, blood cells, and neuronal cells [14]. They are present in most biological fluids, such as plasma, urine, cerebrospinal fluid, and saliva [15]. EVs carry cell origin-specific cargo and are thus considered plausible markers of pathological states if they specifically affect cells [14].

Although the protein profiles of exosomes can vary depending on their cell of origin, some cargo molecules remain constant. Among these are molecules involved in exosome biogenesis (TSG101, VPS26A, VPS29, and VPS35) and intracellular vesicle transport (CD63, CD9, and CD81/CD82). Such molecules are used to identify and validate exosomes in experimental and preclinical studies. Conversely, exosomes harboring more specific cargo, depending on their origin, may be tracked to discover their implications in definite intercellular communications within physiological events and pathological processes [16, 17]. However, exosomes released into biological fluids by various cell types need further study and monitoring for clear identification of their functional significance, while localized intercellular communications present a more obvious model for recognizing the role of EVs as paracrine regulators [18, 19].

In 2008, Al-Nedawi et al. [20] observed exosomes derived from cancer cells and termed them oncosomes. This research group discovered that the commonly mutated variant of the epidermal growth factor receptor variant III (EGFRvIII) was released by glioblastoma cells as EV cargo. They established that only glioblastoma cells representing a highly invasive migratory phenotype secreted EVs harboring EGFRvIII, which activated the pro-oncogenic MAPK and AKT signaling pathways in these cells. Interestingly, cancer cell-derived exosomes were characterized by an elevated diameter reaching 400 nm and the presence of phosphatidylserine on the vesicle surface [21, 22].

Various cancer-related proteins were later isolated from tumor cell-derived exosomes, localized on the exosomal surface, or encapsulated within them. Among these proteins are VEGF, VEGFR, ITGB5, TGF-β1, ADAM10, FAK, and PDL1 [23–25]. Exosome cargo proteins represent enzymes of basic metabolic pathways, molecules stimulating angiogenesis and tumor innervation [26, 27], signaling proteins [28], and immune response modulators [29].

It is well recognized that the reprogramming and redifferentiation signals in cancer cells reflect exosomal cargo and are implicated in cancer cell plasticity that is necessary to maintain the malignant properties of cells and sustain their biological activity in either a dormant (non-proliferative) or proliferative state. Therefore, tumor cell-derived exosomes carry substances that may promote uncontrolled cell division and spread throughout the body. They need to be distinguished from exosomes produced by non-malignant cells, which may promote redifferentiation and reprogramming.

## Cancer cell quiescence reflects its phenotypic plasticity and supports drug resistance

Due to their multi-component nature, exosomes can activate various signaling pathways in recipient cells, altering the intensity of biological processes in these cells [30]. Exosomes’ diverse content and ability to transfer regulatory and structural molecules between cells have induced numerous approaches for their further application as delivery systems for selective cell targeting [31]. However, several limitations such as lack of selectivity, off-target effects, and rapid biodegradation hamper their translation into clinical settings [32].

In addition to properties mentioned above, exosomes have demonstrated an ability to support cancer cell drug resistance, which is highly related to cancer cell plasticity. Phenotypic plasticity is mainly driven by epigenetic mechanisms [33]. In addition to cell reprogramming factors, exosomes can accurately target cancer cells, alter their transcriptional programs, and coordinate cancer invasiveness and metastasis by harboring transcription factors, small non-coding RNA molecules, and other regulatory molecules [34–36].

Tumor dormancy is a reversible state of reduced cell division and metabolic arrest. This condition is supported by mechanisms such as the balance between apoptosis and proliferation and nutrient supply limitations. Transcriptional profiles of non-dividing, quiescent (G0) cancer cells differ from those of actively proliferating [37], reflecting adaptation to varying microenvironments [38]. Hypoxia and serum deprivation induce quiescence [39, 40]. Regulators of quiescence include NR2F1, STAT1, and SMAD1/5 [41–43]. However, the mechanisms driving the re-entry of quiescent disseminated cancer cells into the cell cycle, leading to metastasis, remain less clear.

## TGF-β is a promising target for anti-cancer intervention

TGF-β exerts a pleiotropic role in cell biology, exhibiting dual functional activity as a tumor suppressor in the early stages of carcinogenesis and a tumor promoter in later stages. This duality stems from the complex TGF-β signaling cascade, which encompasses multiple ligand isoforms, heteromeric receptor complexes, and branched intracellular signaling pathways, both canonical and non-canonical [44].

## Main functions of TGF-β in normal cells

In mammals, three main isoforms of TGF-β have been identified: TGF-β1, TGF-β2, and TGF-β3, each exhibiting unique tissue-specific expression patterns and relative abundance. For instance, TGF-β1 is the most prevalent isoform and plays a crucial role in immunoregulation and fibrosis, TGF-β2 is important in the development of the cardiovascular and nervous systems, and TGF-β3 is involved in wound healing and the regulation of cartilage development [45]. Despite similarities in structure and activation mechanisms, these isoforms demonstrate differences in biological activity, highlighting the importance of tissue specificity in TGF-β signaling. TGF-β signaling is mediated by heteromeric receptor complexes consisting of type I (TGFBR1/ALK5) and type II (TGFBR2) receptors, while type III receptors, such as betaglycan, function as co-receptors, modulating the presentation of the TGF-β ligand to the signaling receptors [46]. TGFBR2 is a constitutively active serine/threonine kinase that directly binds TGF-β ligands, initiating a signaling cascade that leads to the recruitment and activation of the type I receptor (TGFBR1) by phosphorylating its GS domain and initiating downstream signal transduction [47]. Intracellular mediators of TGF-β signaling, namely the SMAD family of proteins, are subsequently involved in signal transduction. This family includes receptor-regulated SMADs (R-SMADs), a common mediator SMAD (Co-SMAD), and inhibitory SMADs (I-SMADs). Activated TGFBR1 phosphorylates R-SMADs, such as SMAD2 and SMAD3, inducing a conformational change that allows them to bind to Co-SMAD. Phosphorylated R-SMADs, in turn, bind to SMAD4 and form a heteromeric complex, which then translocates to the nucleus, where it interacts with other transcription factors, co-activators, and co-repressors to regulate the expression of target genes [48]. It is worth noting that the target genes regulated by SMADs are involved in a wide range of cellular processes, including cell cycle control, apoptosis, and even the epithelial-mesenchymal transition (EMT). For example, SMADs regulate the expression of cyclin-dependent kinase inhibitors (CDKIs), such as *p15* and *p21*, leading to cell cycle arrest [49]. SMADs are also capable of regulating the expression of genes involved in apoptosis, such as *BCL2L11* (*BIM*), as well as the expression of transcription factors involved in EMT, *SNAIL*, and *TWIST* [50].

SMAD signaling can be regulated through several distinct mechanisms, including ubiquitination and degradation of SMADs, dephosphorylation by phosphatases, and also by the action of so-called I-SMADs, which block the activation of R-SMADs [51]. Thus, ubiquitination and degradation of SMADs are mediated by the E3 ubiquitin ligases Smurf1 and Smurf2 [52]. Phosphatases, such as PPM1A, dephosphorylate SMADs, resulting in inactivation [51]. I-SMADs, such as SMAD6 and SMAD7, compete with R-SMADs for binding to type I receptors, preventing R-SMAD activation [53].

Regarding non-canonical signaling, it should be mentioned that TGF-β can also activate signaling pathways that are independent of SMADs, including the MAPK pathways (ERK, JNK, p38), the PI3K/AKT pathway, and Rho GTPases (RhoA, Rac1) [54]. For example, TGF-β can activate MAPK pathways, which are known to be involved in cell proliferation, survival, and migration, through various mechanisms, including the activation of small GTPases such as Ras and Rap1 [55]. There is evidence that TGF-β has a multifaceted effect on the PI3K/AKT pathway, which is involved in cell survival and metabolism processes, through both direct and indirect mechanisms mediated by various signaling molecules and GTPases. Activation of PI3K/AKT by TGF-β can be carried out, firstly, through receptor tyrosine kinases (RTKs), which are capable of phosphorylating and activating PI3K, and secondly, through small GTPases, in particular, Ras, which can also trigger the PI3K/AKT cascade. In addition, TGF-β is able to activate Rho GTPases, such as RhoA and Rac1, which leads to the regulation of cytoskeletal changes and cell migration, since RhoA is involved in the formation of stress fibers and ensures cell contractility, while Rac1 promotes the formation of lamellipodia and cell migration [56].

## Disruption of TGF-β signaling in cancer development

Overall, the disruption of TGF-β signaling in cancer is attributed to several well-established mechanisms, including mutations in the genes encoding components of the signaling pathway, such as *TGFBR1*, *TGFBR2*, *SMAD2*, *SMAD3*, and *SMAD4*, frequently observed in various cancer types, including colon cancer, pancreatic cancer, and glioblastoma [57–59]. Imbalances in the expression of TGF-β ligands and their receptors also contribute to cancer progression, with tumor cells potentially overexpressing TGF-β ligands or altering the expression of TGF-β receptors [44]. Given the complex crosstalk between TGF-β signaling and other signaling pathways, such as Ras/MAPK, PI3K/AKT, and Wnt/β-catenin, which are known to regulate processes in carcinogenesis, a number of epigenetic modifications, such as DNA methylation and histone modifications, affect the expression of genes involved in TGF-β signaling through microRNAs and long non-coding RNAs [50]. It should be noted that individual mechanisms, as well as their combination, are associated with the acquisition of an altered phenotype by cancer cells, which is responsible, among other things, for the development of drug resistance, the mechanisms of which we will reveal in more detail in this article.

## Therapeutic strategies for suppressing TGF-β signaling in cancer

Therapeutic strategies targeting TGF-β signaling in cancer encompass approaches based on RNA interference of TGF-β1 and TGF-β2 [60], inhibition of TGF-β signaling via small-molecule inhibitors of TGF-β receptor kinases (TGFBRIs) [61], neutralizing antibodies against TGF-β ligands, and so-called TGF-β trap receptors, which are essentially “decoys” for TGF-β ligands, representing a modified version of the TGF-β receptor or its fragment that exhibits high affinity for TGF-β ligands such as TGF-β1, TGF-β2, and TGF-β3 [62], as well as antisense oligonucleotides [63]. Ongoing clinical trials aim to evaluate the efficacy and safety of various therapeutic strategies targeting the TGF-β signaling pathway, both as monotherapy and in combination with other anticancer treatments. Analysis over the past five years reveals completed clinical trials examining diverse approaches to TGF-β blockade. Specifically, study NCT04551950 assessed bintrafusp alfa—a bifunctional protein combining a TGF-β “trap” with a PD-L1 blocker, in combination with other anticancer therapies in patients with locally advanced or metastatic cervical cancer. Results demonstrated the promise of the combination therapy (https://clinicaltrials.gov/study/NCT04551950). Another study (NCT04574583) explored the combination of SX-682 (a CXCR1/2 chemokine receptor inhibitor) with bintrafusp alfa and CV301 TRICOM (a recombinant poxvirus-based vaccine expressing two tumor-associated antigens MUC1 and CEA, and three costimulatory molecules B7.1, ICAM-1, and LFA-3) in advanced solid tumors, evaluating the safety and antitumor activity of this regimen (https://clinicaltrials.gov/study/NCT04574583). Furthermore, study NCT04247282 evaluated the use of M7824 (anti-PD-L1/TGF-beta “trap”) as monotherapy and in combination with TriAd vaccine and N-803 in patients with resectable squamous cell carcinoma (SCC) of the head and neck (https://clinicaltrials.gov/study/NCT04247282).

Currently, clinical trials are ongoing to further evaluate anti-TGF-β therapy. For instance, study NCT05322408 investigates HCW9218—a bifunctional protein complex designed for the treatment of patients with advanced/metastatic solid tumors (https://clinicaltrials.gov/study/NCT05322408). Additionally, studies NCT04481256, evaluating bintrafusp alfa in combination with chemoradiotherapy in SCC of the esophagus or esophagogastric junction (https://clinicaltrials.gov/study/NCT04481256), NCT06044311, examining vactosertib (a TGF-beta receptor I inhibitor) in combination with standard chemoradiotherapy in adenocarcinoma of the esophagus (https://clinicaltrials.gov/study/NCT06044311), and NCT05400122, assessing NK cells in combination with vactosertib and IL-2 in colorectal cancer (CRC) and hematological malignancies (https://clinicaltrials.gov/study/NCT05400122) are currently enrolling patients.

Future directions include the development of more selective and effective TGF-β inhibitors, the identification of biomarkers for predicting response to TGF-β-targeted therapy, combination therapies, and the investigation of the role of non-coding RNAs and the tumor microenvironment in regulating TGF-β signaling.

Promising treatments may involve the suppression of TGF-β signaling pathway regulators within EVs, particularly in the context of cancer progression associated with drug resistance.

## TGF-β is implicated in the cell-cycle-related phenotype of cancer cells

TGF-β is considered a multifunctional cytokine implicated in several aspects of carcinogenesis. The canonical action of TGF-β is described as bimodal in tumor biology. In normal tissues, TGF-β acts as a tumor suppressor by regulating the cell cycle. Several signal pathways triggered by TGF-β under injury induce cell cycle arrest in the G1 phase. For instance, TGF-β activates CDKIs p21 and p15. Conversely, it has been shown that TGF-β inhibits the oncogene *c-myc*, which favors cell division and represses CDKIs [64]. TGF-β can induce apoptosis by modulating the activity of caspases, anti-apoptotic BCL2, and FAS [65–67].

Conversely, in cancerous tissues, TGF-β supports pro-tumorigenic processes. First, TGF-β released by tumor-associated fibroblasts suppresses the functioning of cytotoxic T-lymphocytes and favors the activity of regulatory T-cells [68, 69]. Second, TGF-β supports EMT, reflecting cancer cell plasticity and phenotype reprogramming [70]. Thus, TGF-β is suggested to play a role in the regulation of cancer cell phenotypic reprogramming and tumor dormancy. Indeed, TGF-β ligands, produced either by tumor or microenvironment cells, modulate dormancy contextually across various cancer models [71]. In particular, TGF-β1 induces the transfer of SCC to a dormant state in vivo [72, 73], whereas the loss of TGF-β R2 in these cells causes augmented tumor growth [74]. Contrarily, the treatment of quiescent T4-2 breast cancer cells in a 3D system with TGF-β1 and ECM protein periostin resulted in increased tumor size, indicating that TGF-β1, together with periostin, facilitates the exit from the quiescent state [71]. Several other studies also reported that TGF-β2 may induce phenotypic switches [41, 75]. Moreover, dormant cells frequently exhibit high levels of TGF-β2 expression compared with their proliferative counterparts [38, 76] suggesting that autocrine TGF-β2 signaling supports the dormant state [75].

TGF-β, like several other cytokines [77], may be present in exosomes produced by cancer cells and transferred from parental to recipient cells, leading to the biological effects mentioned above. Clayton et al. [78] were the first to show the presence of membrane-bound TGF-β1 in exosomes derived from established mesothelioma cells. They demonstrated that these exosomes, also harboring IL-2, were able to stimulate regulatory T-cells and diminish the cytotoxic response of natural killer cells [78]. Another study showed the presence of TGF-β in exosomes derived from murine adenocarcinoma cells. These aforementioned EVs stimulated the differentiation of bone marrow myeloid cells into pro-tumorigenic myeloid-derived suppressor cells, followed by their enhancement of tumor growth [79]. TGF-β originating from head and neck carcinoma cells-derived EVs stimulated macrophage activation and their reprogramming into a pro-angiogenic phenotype that further promoted tumor vascularisation in vivo [80]. Another study noticed that exosome-derived TGF-β1 was characterized by higher signaling capacities compared with free TGF-β1. Both active and latent membrane-bound TGF-β1 demonstrated the ability to transfer into recipient human mesenchymal stem cells (MSCs), enhancing their migratory capacities via SMAD-dependent pathways [81].

Not only TGF-β itself but also its receptors can be carried in exosomes. Thus, it has been reported that breast cancer cells-derived exosomes contain an active form of TGF-β type II receptor (TβRII) in their cargo, promoting the activation of TGF-β signaling in recipient cells. Interestingly, the uptake of exosomal TβRII by tumor cells with a low aggressiveness phenotype initiated EMT that resulted in these cells acquiring the features of cancer stem cells and facilitated the increasing number of metastases in animal models. In parallel, TβRII transferred to CD8^+^ T-cells via exosomes activated the transcriptional modulator SMAD3, which corresponded to diminished CD8^+^ T-cell functioning and led to ineffective immunotherapy [82].

Conversely, exosomes derived from tumor-associated macrophages led to increased expression of TGF-β signaling pathway activators in meningioma cells, specifically *TGFBI*, *SNAI1*, *MMP2*, *TGF-β1*, *TGF-β2*, *IGFBP7*, and *LTBP2*, leading to tumor cells obtaining a more aggressive phenotype [83]. Meanwhile, TGF-β signaling activation was determined only in breast cancer cells with an aggressive phenotype and corresponded to elevated EV secretion and rapid tumor progression in animal models. In turn, the inhibition of EV release in highly aggressive breast cancer cells diminished TGF-β signaling activation and reduced their invasive phenotype, impairing tumor progression and the onset of metastasis. Thus, a new approach to regulate breast cancer dissemination was presented, involving the inhibition of exosome trafficking together with blocking TGF-β signaling in phenotypically aggressive breast cancer in vitro and in vivo [84].

Summarizing, these studies demonstrate the impact of exosomes on TGF-β functioning, which is crucial to regulating for managing tumor progression. EVs could be a plausible target for maintaining a low-aggressive phenotype of cancer cells, thus lowering tumor dissemination. However, to implement such an approach, delicate monitoring of EV cargo and their trafficking is needed. TGF-β, delivered both directly through exosomes and produced or activated locally in response to signals potentially triggered by exosomes, leads to diverse alterations within normal and cancerous tissues.

## Exosomal TGF-β functions

### The role of cancer-associated fibroblasts and TGF-β in the tumor microenvironment

Activated fibroblasts in the tumor microenvironment are known as cancer-associated fibroblasts (CAFs) [85]. Cancer cells attract CAFs largely via regulation by growth factors secreted by primary tumor cells and immune elements. Specifically, TGF-β, PDGF, and FGF-2 play key roles in the activation of fibroblasts during acute and chronic tissue injuries, as well as in their repair [86]. Studies on the impact of the ECM stiffens that is important for metastasis development, have shown that TGF-β enhances the activity of lysyl oxidase (LOX), an enzyme responsible for collagen cross-linking and remodeling [87]. The mechanism of fibroblast activation remains unclear; however, it is known that the phenotype and behavior of cancer cells can be altered through modifications of the ECM [88], indicating that tumor cells do not function autonomously but heavily rely on signals and the composition of the ECM in their microenvironment. TGF-β can also alter the tumor cell microenvironment by mobilizing various cell types, including fibroblasts and immune cells. This helps create favorable circumstances for the awakening and division of quiescent cells.

### Exosomes as mediators of TGF-β-dependent fibroblast differentiation and tumorigenesis

Notably, exosomes derived from prostate cancer cells trigger TGF-β1-dependent fibroblast differentiation, forming a myofibroblast phenotype similar to stromal cells isolated from prostate cancer tissue. Additionally, exosomes enhanced angiogenesis in vitro and promoted tumor growth in vivo. Meanwhile, myofibroblasts generated under soluble TGF-β1 stimulation did not show proangiogenic or tumorigenic capacities [89].

Exosomes released by bladder cancer cells are internalized by fibroblasts and promote proliferation and enhanced expression of CAF markers. These cells contained TGF-β followed by activation of SMAD-dependent signaling. Application of TGF-β inhibitors diminished the expression of CAF-associated proteins in healthy fibroblasts [90].

Gastric cancer exosomes trigger the differentiation of umbilical cord-derived MSCs into CAFs through exosome-mediated TGF-β trafficking and activation of the TGF-β/SMAD pathway, which may represent a novel mechanism for the transition of MSCs to CAFs in cancer [91].

Elevated levels of membrane-bound TGF-β were determined in EVs released by metastatic osteosarcoma cells. TGF-β interaction with its receptor on the surface of MSCs resulted in increased synthesis and release of pro-inflammatory IL-6, which supported the metastatic process by immunosuppression [92].

### The role of exosomal non-coding RNAs in regulating TGF-β signaling

Several studies have elucidated the role of EV-derived non-coding RNAs on TGF-β expression with subsequent effects on TGF-β signaling in recipient cells. For instance, colorectal carcinoma cells release EVs containing non-coding RNA circPAGRGL, which induces TGF-β expression, followed by enhanced cell proliferation and metastasis development. Additionally, EVs supported the phenotypic switch of oncosuppressive N1 neutrophils to pro-tumorigenic N2 by activating miR-142-3p and miR-506-3p [93]. Gastric cancer cell-derived exosomes containing miR-21-5p showed the ability to promote the acquisition of mesenchymal features by peritoneal mesothelial cells. This stimulated invasion by activating tumor TGF-β signaling pathways via inhibiting SMAD7, which acts as a TGF-β suppressor [94].

Furthermore, the surface components of EVs were shown to interact with TGF-β, bringing combined signals into recipient cells. This phenomenon was observed in MSCs, stimulating them to secrete IL-6 to support a pro-inflammatory microenvironment [95]. Chronic myeloid leukemia cell-derived exosomes induced transcriptional reprogramming in bone marrow MSCs, affecting mRNA levels of several proteins involved in leukemia cell development. Among them, TGF-β levels were diminished under treatment due to the ability of TGF-β to induce the differentiation and suppress proliferation of leukemic cells. At the same time, the treatment of bone marrow MSCs and macrophages with chronic myeloid leukemia cell-derived exosomes altered the balance between pro- and anti-oxidative processes in these cells. Increased production of NO observed in bone marrow MSCs may favor the establishment of an immunosuppressive environment and stimulate angiogenesis. Conversely, decreased NO levels observed in macrophages may correspond to alterations in their polarization, shifting from M1 to M2, supporting a pro-tumorigenic environment as well [96].

## EVs and TGF-β in premetastatic niche formation and organ remodeling

**Figure 1:**
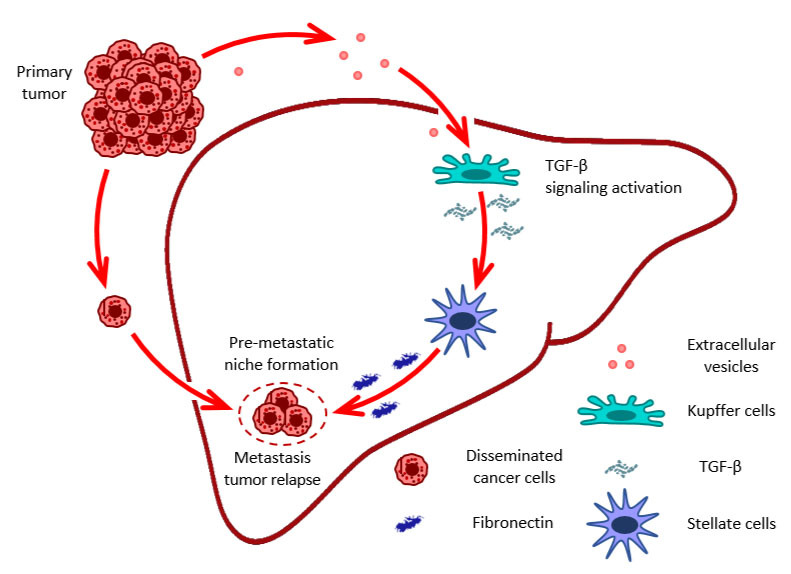
**EVs role in pre-metastatic niche formation in distant organs and tumor relapse.** Figure shows fibronectin as important component of the ECM that is involved in the formation of the premetastatic niche. ECM: extracellular matrix; EVs: extracellular vesicles; TGF-β: transforming growth factor-β

It is well established that metastasis development is organ-specific, and tumor-disseminated cells mostly reside in favored sites. However, EVs released by primary tumor cells may reach these organs to precondition the environment for further disseminated cancer cells residing there. Thus, EVs may be involved in the fate of cancer disease progression by organ remodeling [97]. In particular, EVs produced by pancreatic ductal adenocarcinoma remodeled liver tissue, making it more favorable for metastasis development. Uptake of EVs by hepatic Kupffer cells resulted in TGF-β signaling pathway activation and elevated TGF-β release, which, in turn, stimulated stellate cells to produce fibronectin [98] (Figure 1).

ECM interaction with tumor cells residing quiescent in distant organs is an important feature determining tumor progression and relapse. The ECM dynamically regulates the state of tumor cells, while fibronectin supports their quiescent status [99]. Another study showed that EVs produced by bone marrow-derived cells in a lung cancer murine model contain miR-92, which also through TGF-β signaling activation in liver stellate cells, induced the production of collagen type I. Thus, miR-92 transferred via EVs to liver stellate cells decreased *SMAD2* expression and activated TGF-β signaling, promoting rapid liver metastasis development [100].

## TGF-β and EMT: implications for tumor progression and metastasis

Phenotypic plasticity is considered a specific feature of cancer cells. Both genetic and non-genetic alterations support the acquisition of new biological characteristics by cancer cells to avoid injurious stimuli. Vascular mimicry, as well as cancer cell dedifferentiation and mutational instability, represent mechanisms that support cancer cell reprogramming. EMT is another mechanism by which cancer cells may alter their phenotype. Recent studies suggested that mesenchymal features of cancer cells can shift to epithelial cells as well [101]. Moreover, it has become clear that while EMT is a necessary event in cancer stem cells for the onset of the metastatic process, EMT is required for further metastasis development. Thus, E-selectin stimulates keratin-14 expression in prostate cancer cells, followed by the activation of Wnt signaling and engaging metastasis in bone tissue [102]. TGF-β is a well-established enhancer of EMT, a process in which epithelial cells acquire a more motile and invasive mesenchymal phenotype that promotes metastasis. This is especially important for quiescent cells re-entering the cell cycle, as it ensures their ability to migrate and form metastatic nodes.

It has been reported that exosomes derived from dormant lung adenocarcinoma A549 cells could be taken up by fibroblasts. Exosomal ITGB6 transferred into fibroblasts induced their transition to CAFs via activating the TGF-β pathway. Thus, high ITGB6 expression indicated TGF-β pathway activation and ECM remodeling [103].

TGF-β1 levels were found to be increased in CAFs compared with the levels in normal omental fibroblasts. Therefore, both TGF-β1 treatment or treatment with exosomes derived from CAFs affected ovarian cancer cells to achieve spindle-cell morphology, form protrusive structures, enhance migrative and invasive capacities, and induce mesenchymal marker N-cadherin and vimentin expression mediated by SMAD2/3 signaling. Conversely, blocking TGF-β1 depleted the aforementioned alterations, confirming the ability of TGF-β1 to regulate both processes, including EMT [104].

## Immunosuppressive effects of TGF-β and therapeutic implications

However, TGF-β can suppress the immune response by acting on various factors of the immune system. In terms of dormancy, it allows quiescent cells to evade immune surveillance and supports the survival of slow-cycling/non-cycling cancer cells for long periods for their subsequent reactivation later.

Exosomes derived from renal cell carcinoma have been shown to contain TGF-β1 and transfer it to tumor-infiltrating natural killer cells, impairing their functioning through activation of the TGF-β/SMAD pathway [105]. Exosomes produced by head and neck SCC (HNSCC) cell lines contain TGF-β and stimulate macrophage chemotaxis without a detectable M1/M2 phenotype shift and reprogramme primary human macrophages into a proangiogenic phenotype characterized by upregulation of proangiogenic factors and functions [80].

We have summarized all properties of exosomal TGF-beta in a table, characterizing its main regulatory properties related to carcinogenesis (Table 1).

**Table 1:** Key functions of exosomal TGF-β

**Function of exosomal TGF-β**	**Mechanism**	**Resulting effect**	**Cancer type(s)**	**Reference(s)**
Fibroblast differentiation and activation	Activates SMAD-dependent signaling upon internalization into fibroblasts.	Promotes differentiation of fibroblasts into CAFs, enhances CAF marker expression.	Prostate cancer, bladder cancer, gastric cancer, and lung adenocarcinoma.	[89–91, 103]
ECM remodeling	Enhances LOX activity, leading to collagen cross-linking.	Stiffens the ECM, creating a favorable environment for metastasis development.	General (tumor microenvironment).	[87, 103]
Angiogenesis promotion	Stimulates angiogenesis via myofibroblast phenotype.	Enhances tumor growth in vivo.	Prostate cancer.	[89]
MSC modulation	TGF-β interaction with receptors on MSCs.	Increases synthesis and release of pro-inflammatory IL-6, supporting metastasis via immunosuppression.	Osteosarcoma, chronic myeloid leukemia.	[92, 95, 96]
Non-coding RNA-mediated signaling	Delivery of circPAGRGL, miR-21-5p, miR-92, miR-142-3p, and miR-506-3p via exosomes.	Induces TGF-β expression, enhances cell proliferation and metastasis, promotes EMT, and supports phenotypic switch of neutrophils to pro-tumorigenic N2.	Colorectal cancer, gastric cancer, and lung cancer.	[93, 94, 100]
Premetastatic niche formation	Activation of TGF-β signaling in hepatic Kupffer cells and stellate cells.	Remodels liver tissue, making it more favorable for metastasis by stimulating fibronectin and collagen type I production.	Pancreatic ductal adenocarcinoma, lung cancer.	[98, 100]
EMT induction	Activation of SMAD2/3 signaling.	Promotes a more motile and invasive mesenchymal phenotype, enhancing migration and invasion.	General (ovarian cancer).	[104]
Immune suppression	Transfer of TGF-β1 to tumor-infiltrating natural killer cells.	Impairs NK cell functioning via TGF-β/SMAD pathway activation, supports immune evasion of quiescent cells.	Renal cell carcinoma.	[105]
Macrophage reprogramming	Stimulation of macrophage chemotaxis and reprogramming.	Promotes proangiogenic phenotype in macrophages, characterized by upregulation of proangiogenic factors.	Head and neck squamous cell carcinoma.	[80]

CAFs: cancer-associated fibroblasts; ECM: extracellular matrix; EMT: epithelial-mesenchymal transition; LOX: lysyl oxidase; MSC: mesenchymal stem cell; TGF-β: transforming growth factor-β

Additionally, the combinatorial treatment by TGF-β inhibitors with existing immunotherapeutic agents has demonstrated promising outcomes. Nonetheless, this therapeutic approach necessitates meticulous evaluation and patient stratification to maximize treatment efficacy. Within this context, it is necessary to identify endogenous delivery pathways of TGF-β to modulate their regulatory mechanisms on cancer cells and the tumor microenvironment.

## TGF-β-mediated cancer cell plasticity leads to drug resistance

Drug resistance is a major challenge in modern oncology. Numerous mechanisms have been associated with this phenomenon, including increased expression of drug efflux transporters, activation and reactivation of proliferative signaling, evasion of pro-apoptotic stimuli, mutational heterogeneity, and immune escape [106–110]. However, the activation of TGF-β signaling has been shown to correspond with drug resistance in various cancer types, such as melanoma [111], non-small cell lung cancer (NSCLC) [112], breast cancer [113], hepatocellular carcinoma (HCC) [114], CRC [115], SCC [73], osteosarcoma [116], and in tumor-initiating cells of some cancer types [44, 117]. In addition, high levels of TGF-β in patients with breast cancer, NSCLC, HCC, and CRC are associated with poor prognosis [118–122]. Tan et al. [123] discovered that exosome-mediated TGF-β1 transfer affects the sensitivity of cancer cells to therapy. In their study, exosomes were isolated from both adriamycin-sensitive and -resistant breast cancer cells, followed by the determining exosomal cytokine levels. Cells resistant to adriamycin treatment were characterized by increased TGF-β1 expression. Moreover, exosome-mediated intercellular transfer of TGF-β1 resulted in the phosphorylation of SMAD2 activation and enhanced cell survival by repressing apoptosis and increased cell mobility in a zebrafish breast cancer xenograft model [123].

Besides its implication in EMT acting to protect from injury induced by anti-cancer agents, TGF-β supports autophagy via gene expression regulation. Hence, the treatment of MSCs, a component of the osteosarcoma microenvironment, with leptin, a regulator of osteoblastogenesis characteristic of metastasis in bone cancer, stimulated autophagy and activated TGF-β signaling that corresponded to cisplatin resistance in osteosarcoma cells [124]. Similarly, in breast and pancreatic cancer cell lines, TGF-β signaling via EMT was shown to support cisplatin resistance by increasing the expression of deubiquitinase USP27X, which, in turn, increases the stability of the EMT-associated protein SNAIL1 [125].

Oshimori et al. [72] developed a system for in vitro/in vivo imaging of TGF-β signaling that allowed the observation of a heterogeneous pattern of SMAD2/3 expression activated by TGF-β in SCC cells. SCC cells that responded to TGF-β were slowly cycling cells, while non-responders were actively proliferating cells. Meanwhile, these slow-cycling cells were characterized by resistance to cisplatin. Further analysis identified the overexpression of the redox-sensitive transcription factor NRF2 and p21, cyclin-dependent kinase 1A, which is specific for non-cycling (quiescent or senescent) cells. Thus, the activation of antioxidant factors mediated by TGF-β can be a mechanism by which cancer cells residing in the resting phase of the cell cycle may resist anti-cancer treatment [72]. Besides, in SCC cells, TGF-β can activate the AKT pathway, followed by overexpression of *SOX2* and *ABCG2*, which are characteristic of cancer stem cells, indicating the ability to induce cancer cell dedifferentiation that corresponds to cisplatin resistance [126].

On the other hand, several studies have shown that TGF-β pathway downregulation can also be associated with chemoresistance [16, 127, 128]. For instance, the inhibition of the transcription factor MITF together with TGF-β signaling has been shown to increase resistance to MEK inhibitors in melanoma cells [129]. Moreover, *SMAD2* downregulation or the loss of *SMAD4* diminishes TGF-β-dependent expression of tumor suppressor genes, leading to the upregulation of anti-apoptotic proteins BCL2 and BCLw, corresponding to resistance development of NSCLC cells and CRC cells to platinum anti-cancer compounds and 5-fluorouracil, respectively [128, 130].

In summary, the intricate interplay between TGF-β signaling and drug resistance mechanisms is evident across a spectrum of cancer types. This association is not unidirectional, as both the activation and downregulation of TGF-β pathways have been implicated in conferring resistance to various chemotherapeutic agents. A concise overview of these associations, highlighting the cancer type, the drug involved, and the specific TGF-β signaling component implicated in resistance, is presented in the table (Table 2).

**Table 2:** Association of TGF-β signaling with drug resistance across various cancer types

**Effect of TGF-β signaling**	**Mechanism of drug resistance**	**Cancer type(s) implicated**	**Reference(s)**
Overall association	Activation of TGF-β signaling often correlates with increased drug resistance.	Melanoma, non-small cell lung cancer, breast cancer, hepatocellular carcinoma, colorectal cancer, squamous cell carcinoma, osteosarcoma, and tumor-initiating cells.	[44, 73, 111–116]
Exosome-mediated transfer	Exosome-mediated transfer of TGF-β1 from resistant cells increases cell survival, reduces apoptosis, and enhances cell mobility.	Breast cancer.	[123]
Autophagy promotion	TGF-β promotes autophagy, leading to increased drug resistance.	Osteosarcoma (specifically, in mesenchymal stem cells), breast and pancreatic cancer cell lines.	[25, 124]
Quiescence/senescence	TGF-β activation leads to a population of slow-cycling cells resistant to cisplatin, potentially via NRF2 and p21 overexpression.	Squamous cell carcinoma.	[72]
Cancer stem cell induction	TGF-β can activate the AKT pathway, leading to increased expression of cancer stem cell markers (SOX2, ABCG2) and cisplatin resistance.	Squamous cell carcinoma.	[126]
Pathway downregulation	Downregulation of TGF-β signaling (e.g., MITF inhibition, SMAD2 downregulation, and SMAD4 loss) can paradoxically lead to drug resistance by upregulating anti-apoptotic proteins.	Melanoma, non-small cell lung cancer, and colorectal cancer.	[128–130]

TGF-β: transforming growth factor-β

## Strategies to inhibit TGF-β signaling by blocking the spread of EVs

Despite considerable progress in the implementation of novel anti-cancer approaches into the clinic, cancer remains a major health problem due to rapidly developing drug resistance driven by the high plasticity and heterogeneity of cancer cells. As revealed in numerous studies, TGF-β signaling is implicated in the development of cancer treatment insufficiency via various pathways. Although the application of TGF-β inhibitors failed to provide evident therapeutic impact, it does not negate the multiple effects of TGF-β signaling in cancer progression and drug resistance. However, combinatorial treatment regimens have been reported to enhance the efficacy of TGF-β inhibitors. For instance, their application with immunotherapy has been shown to yield greater results in several preclinical studies [131].

However, TGF-β itself may support both cancer cell plasticity and heterogeneity, where TGF-β or its regulatory molecules via EVs provide intercellular communication resulting in a phenotypic switch of target cells. TGF-β is crucial for cancer cell communication with the ECM and microenvironment structures. EVs containing integrins αν5 interact with liver cells, while EVs loaded with integrins α6β4 and α6β1 demonstrate intensive binding to lung fibroblasts and epithelial cells [23]. CRC cell-derived EVs containing integrin β-like 1 (ITGBL1) via the bloodstream reach fibroblasts and stellate cells in the lungs and liver. The latter starts to secrete TGF-β and pro-inflammatory cytokines IL-6 and IL-8, leading to further tumor progression and dissemination [132]. This poses the question of whether selectively blocking not TGF-β signaling directly but its activators may be an efficient approach to regulating cancer dissemination. Indeed, exosomes derived from MSCs and loaded with miR-200a mimic abrogated TGF-β-activated EMT in gastric cancer cells, which was represented as upregulation of E-cadherin expression and downregulation of β-catenin, vimentin, ZRB1, and SNAIL1 expression. The authors presume that EMT markers depression corresponds to hampering further metastasis development [133]. TGF-β stimulated PD-L1, an immune-suppressive antigen, loading in breast cancer cell-derived exosomes, followed by CD8^+^ cells dysfunction. TGF-β inhibitor application significantly reduced PD-L1 presence in EVs. Moreover, neutralizing PD-L1 harboring exosomes together with TGF-β blockage led to effective killing of breast cancer cells by T-cells [134]. Therefore, targeting exosomes derived from specific cells using antibody fragments or peptides represents a promising direction in biomedical research and therapy. The surface antigens of exosomes can be modified by attaching specific ligands, enabling the delivery of therapeutic agents directly to target cells.

Restoring TGF-β signaling in quiescent SCC cells by applying the DNA methylation inhibitor 5-azacytidine with the ligand of the retinoic acid receptor supported the existence of the aforementioned cells in a quiescent state, which delayed tumor relapse and metastasis development [135]. This study underscores that effective manipulation with TGF-β or with components of its signaling can be achieved by selective targeting depending not only on the nature of the cells but also on their phenotype. The necessity of quiescent cancer cell therapeutic targeting is still obscure and under intensive study now. However, TGF-β signaling can be a putative target to control cancer cell plasticity.

## Conclusions

**Figure 2:**
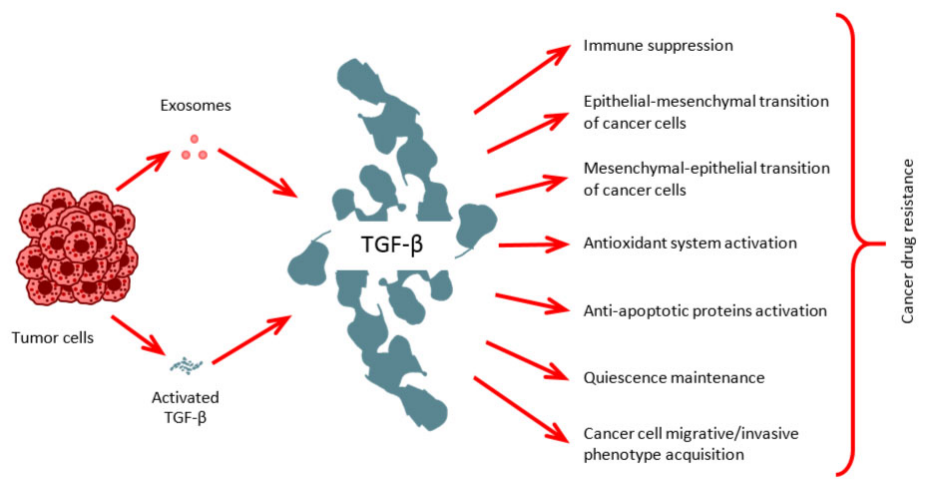
**TGF-β role in carcinogenesis.** Cancer cells release TGF-β directly and secrete it as EV cargo to promote numerous cancer-related processes.

In conclusion, exosomes represent a multifaceted and dynamic mechanism employed by cancer cells to achieve phenotypic reprogramming. The capacity of EVs to shuttle biological information, both locally and distally, significantly influences cancer progression, although potential oncosuppressive roles warrant further investigation. While TGF-β traditionally exhibits anti-cancer properties in normal cells, facilitating cell cycle exit and apoptosis of compromised cells, its role in preventing malignant transformation deserves greater emphasis. Furthermore, modulating cancer cell phenotypic plasticity via TGF-β related signals constitutes a potential avenue for circumventing drug resistance. Accumulating evidence highlights the critical role of EVs in mediating TGF-β signaling, either through the direct delivery of TGF-β itself or, more frequently, by transporting regulatory molecules that modulate TGF-β activation within target tissues. Specifically, EVs can deliver integrins, cytokines, and microRNAs that influence the tumor microenvironment and promote tumor progression (Figure 2). Ongoing research focused on deciphering the mechanisms of intercellular communication via EVs in the context of TGF-β-regulated cancer cell plasticity is poised to yield more precise and effective therapeutic strategies, potentially targeting specific EV subsets or interfering with their cargo-mediated effects on TGF-β signaling in recipient cells.

## Abbreviations

CAFs: cancer-associated fibroblasts
CDKIs: cyclin-dependent kinase inhibitors
Co-SMAD: common mediator SMAD
CRC: colorectal cancer
ECM: extracellular matrix
EGFRvIII: epidermal growth factor receptor variant III
EMT: epithelial-mesenchymal transition
EVs: extracellular vesicles
HCC: hepatocellular carcinoma
I-SMADs: inhibitory SMADs
MSCs: mesenchymal stem cells
NSCLC: non-small cell lung cancer
R-SMADs: receptor-regulated SMADs
SCC: squamous cell carcinoma
TGF-β: transforming growth factor-β
TβRII: TGF-β type II receptor

## Declarations

### Copyright

© The Author(s) 2025.
